# Building a Phylogenomic Pipeline for the Eukaryotic Tree of Life - Addressing Deep Phylogenies with Genome-Scale Data

**DOI:** 10.1371/currents.tol.c24b6054aebf3602748ac042ccc8f2e9

**Published:** 2014-04-02

**Authors:** Jessica R. Grant, Laura A. Katz

**Affiliations:** Department of Biological Sciences, Smith College, Northampton, Massachusetts, USA; Department of Biological Sciences, Smith College, Northampton, Massachusetts, USA

## Abstract

Background Understanding the evolutionary relationships of all eukaryotes on Earth remains a paramount goal of modern biology, yet analyzing homologous sequences across 1.8 billion years of eukaryotic evolution is challenging. Many existing tools for identifying gene orthologs are inadequate when working with heterogeneous rates of evolution and endosymbiotic/lateral gene transfer. Moreover, genomic-scale sequencing, which was once the domain of large sequencing centers, has advanced to the point where small laboratories can now generate the data needed for phylogenomic studies. This has opened the door for increased taxonomic sampling as individual research groups have the ability to conduct genome-scale projects on their favorite non-model organism. Results Here we present some of the tools developed, and insights gained, as we created a pipeline that combines data-mining from public databases and our own transcriptome data to study the eukaryotic tree of life. The first steps of a phylogenomic pipeline involve choosing taxa and loci, and making decisions about how to handle alleles, paralogs and non-overlapping sequences. Next, orthologs are aligned for analyses including gene tree reconstruction and concatenation for supermatrix approaches. To build our pipeline, we created scripts written in Python that integrate third-party tools with custom methods. As a test case, we present the placement of five amoebae on the eukaryotic tree of life based on analyses of transcriptome data. Our scripts available on GitHUb and may be used as-is for automated analyses of large scale phylogenomics, or adapted for use in other types of studies. Conclusion Analyses on the scale of all eukaryotes present challenges not necessarily found in studies of more closely related organisms. Our approach will be of relevance to others for whom existing third-party tools fail to fully answer desired phylogenetic questions.

## Introduction: Setting the Stage

Phylogenomic analyses can be used for many purposes, from shedding light on the ancient innovations that created the diversity we see today to understanding the function of organisms in varying ecosystems, and even the very practical study of epidemiology and emerging diseases. Phylogenomics can also be used to place unstable or newly discovered taxa on the tree 1 ^,^ 2 and to understand the relationships among organisms that have been difficult to study with more traditional methods (i.e. amoeboid taxa with few morphological characters 3 ^,^ 4 ). Given the age and tremendous diversity within many clades, large-scale gene- and taxon-rich analyses help us discern the structure of the tree 5 ^,^ 6 as well as address the most ancient innovations, such as the root of all eukaryotes^7^.

While there are many useful phylogenomic tools available 8 ^,^ 9 ^,^ 10 ^,^ 11 ^,^ 12, we have found that they are not designed to capture the tremendous complexity of orthologs that exist in lineages that span the ~1.8 billion years since the origin of eukaryotes. Analyses of eukaryotes come with unique challenges, such complex patterns of paralogy, incongruence due to endosymbiotic/lateral gene transfer, and heterogeneous rates of evolution in many lineages. Also, since we are aiming to maximize the depth and evenness of taxon sampling across the eukaryotic tree of life, it is critical that we be able to curate taxon names and maintain a taxonomically informed dataset. Further, we wanted to incorporate Guidance^21^ into our pipeline as this alignment tool allows iterative removal of poorly aligned taxa followed by masking of poorly aligned characters. Incorporating Guidance into our custom-built pipeline generated robust multi-sequence alignments for gene tree and supermatrix analyses.

As the cost of genome-scale sequencing continues to fall, the bottleneck to scientific discovery is shifting from data acquisition to the organization and analysis of high throughput data. Many laboratories have developed pipelines for their own use, and we recognize that everyone will have to approach their analyses in their own way, which may mean adapting or modifying scripts. However, we believe that presenting our pipeline and discussing the problems we encountered along the way will help others to benefit from the lessons we have learned and to avoid some of the same pitfalls.

We divide this manuscript into three sections. First we provide an overview and discuss some of the major insights from our pipeline development including how to: approach taxon sampling; curate and organize large-scale data; mine large databases; and refine the data into a tractable dataset. In the second section, we describe in more detail the innovations in the pipeline and the function of the Python scripts, including where other users might need to modify parameters for their specific questions. Finally, we present a case study that uses the pipeline to analyze the transcriptomes of five amoeboid species with the aim of placing them on the eukaryotic tree of life.

## Part I: A general overview of our methods

Though the unique questions being addressed in individual studies will dictate the choice of taxa and markers, there are some issues that all groups using high-throughput data for evolutionary analyses will encounter. These common issues are outlined here, along with an overview of our Python scripts. Part II (below) includes a more detailed description of our Python scripts and how they might be modified to suit a different user’s needs.

**Taxon and gene sampling – our approach**

When first approaching large-scale analyses, the challenges can be overwhelming, including knowing which taxa to include and which markers to use. We are interested in the eukaryotic tree of life, which spans approximately 1.8 billion years 13 ^,^ 14 , so choosing appropriate genes and taxa that represent as many of the clades of eukaryotes as possible is essential. In addition, non-vertical inheritance (i.e. lateral gene transfer) can best be understood in the context of the tree of all life, so we also included diverse bacterial and archaeal lineages. The pipeline is not limited to the deep phylogenetic analyses that we have used it for, and could be adapted to be used with greater sampling of a smaller taxon set, for example, looking for orthologs in all strains of a bacterial species.

Our first step in choosing loci was to identify an appropriate external source for orthologous clusters of genes. We examined several databases that cluster sequence data into orthologous groups and found that for depth of sampling in eukaryotes and accurate clustering, the database of orthologous groups from OrthoMCL (http://www.orthomcl.org/) best suited our needs. For comparison, the InParanoid dataset 15 clusters data from only 26 organisms, mostly multicellular eukaryotes, and the eukaryotes in ncbi’s COG have not been updated since 2003. OrthoMCL, originally developed by the *Plasmodium *community 16 , currently includes 124,740 orthologous groups sampled from 98 eukaryotes plus 44 bacteria and 16 archaea (version 5; http://orthomcl.org/). We then identified genes in OrthoMCL DB that were found in at least four of the five putative major eukaryotic clades (i.e. Amoebozoa, Excavata, Opisthokonta, Plantae, and SAR) and that had a reasonable (e.g. ≥49 out of the maximum of 98) sampling from eukaryotes in OrthoMCL. This yielded a starting point of 1668 genes, of which 1554 were retained for phylogenomic analyses at the end of our pipeline (>500,000 characters). An alternative to using OrthoMCL DB would be creating a custom database from a BLAST all against all script, but BLAST is difficult to parameterize to avoid false positives (e.g. genes that share only a common motif or functional domain) while still capturing the heterogenous patterns over ~1.8 billion years of evolution 13 ^,^ 14 ^,^ 17 . A BLAST all against all strategy may work well for those working on a smaller time scale.

Similar challenges exist when choosing taxa, as the goal is often to maximize inclusion while maintaining limits that allow for efficient downstream analyses. Given our interest in diverse eukaryotes, we selected all eukaryotes that had 100 or more protein sequences available in GenBank, with limits on the numbers of plants, animals and fungi to keep the size of the dataset manageable. For well-sampled microbial genera (e.g. Plasmodium, *Trypanosoma, Phytophthora*) we generally restricted ourselves to two species per genus. To include outgroups and potential donors for LGTs, we also included representative bacterial and archaeal genomes from GenBank, though these data were not used in all analyses.

**Curating data for ease of use**

Keeping track of the names of sequences in large-scale data is critical and takes forethought. Many third party software packages demand sequence names be a certain length and without certain symbols. Some will truncate sequence names causing endless headaches down the road when attempting to interpret results. At the same time, simply numbering sequences makes it difficult to quickly assess the intermediate output of the analyses (e.g. alignments, single gene trees). We have found that the best way to name sequences is a combination of standardized names and numbers. Our standard naming system for all taxa includes a short code for each major clade, subclade, and taxon, plus a unique identifier for each sequence. For each taxon, we use two letters denoting the major clade of eukaryotes, two letters denoting the subclade and four letters that uniquely identify that taxon. For example, *Homo sapiens *sequences are labeled Op_me_hsap (**Op**isthokont, **me**tazoa, ***H*** *omo* ***sap*** *iens*). This standard makes scanning alignments and trees for misplaced taxa easy. Following these standardized taxon names come a number unique to each sequence, which may be a GI number or a code from our own transcriptome data. At points in our pipeline (i.e. prior to running Guidance, Mafft), it was necessary to number each sequence because of the problem of name truncation; in these instances, the scripts assign a number to each sequence and keep a key to rename the sequences back when the relevant phase of the analyses are complete.

**Mining and refining public data for orthologs**

We used BLAST 18 as a rapid first pass in order to pull large amounts of sequence data from each taxon and the resulting files were later refined through more sophisticated (but time intensive) methods as described below. BLAST is currently the most common way of assessing homology in the rapidly-growing public data and it often works well despite being a fairly blunt tool. Yet, with scores such as e-value and bit-score that depend on database size and sliding windows, BLAST is hard to parameterize to return just the orthologs of genes of interest. Also, BLAST can return sequences that are not useful for phylogenetic analyses, including sequences that are identical or so similar that they do not add information to the analysis but instead make the final analysis more time-consuming (e.g. allelic variation). BLAST can also capture sequences that share a motif with the gene of interest but are not clearly homologous. Hence, we used BLAST to find potential homologs but then used fast tools (e.g. pairwise alignments from Needle, guide trees built in Mafft) to remove too-similar sequences quickly in order to save computational time, and to remove non-homologous sequences that can create problems during later steps such as alignment building. How these tools were used is described in greater detail in the next section.

To assess our approach, we compared our orthology detection against KEGG (http://www.genome.jp/kegg/), which includes an overlapping taxon set with our pipeline. For five genes, we assessed the orthologs identified by our pipeline for two taxa found in both OrthoMCL DB and KEGG (*Homo sapiens *and *Arabidopsis thaliana*) and for two taxa found in KEGG and added by our pipeline (*Paramecium tetraurelia *and *Phytophthora infestans*). For*H. sapiens *and *A. thaliana*, OrthoMCL DB included all the sequences found in KEGG, or more. Where OrthoMCL identified more sequences, these tended to be closely related in-group paralogs that were removed in later steps of our pipeline (see Supplemental Table S1). For *P. tetraurelia *and *P. infestans*, our pipeline returned all the sequences found by KEGG, except in one case where KEGG included two sequences that were identical except for a short indel. For one gene, our pipeline returned 9 sequences for Paramecium where only two were found in KEGG. Again, these nine included in-group paralogs that were removed in later steps of our pipeline (see Supplemental Table S1).

Another type of data that may be returned by BLAST, especially from mining EST or other short read databases, are sequence fragments from a single taxon that do not overlap with one another. As described in more detail below, if there were multiple non-overlapping sequences from the same taxon, we removed all but the longest sequence. Since the determination of paralogs can be critical in phylogenetic analyses, we took a conservative approach to retain only convincing paralogs. In essence, our script kept sequences that overlap substantially with the longest sequence in each taxon, and that were neither too similar (e.g. >97% identity) nor too different (i.e. as determined by pairwise alignment; see below for details). We took this approach as there is no way to know if two non-overlapping sequences represent different regions of the same locus, or if they represent paralogous loci.

**Dealing with contamination**

Misidentified sequences are found frequently on GenBank, especially from large-scale sequencing projects and from studies of non-model organisms where an unusual sequence might not be recognized as coming from another taxon (e.g. as a contaminant from the environment or food source). Our approach was to remove single sequences that cluster within distantly-related clades in phylogenetic trees, for example, a eukaryotic taxon with a sequence that clusters within the archaea or bacteria. While these sequences might represent recent lateral gene transfers (i.e. involving transfers into only a single taxon in dataset), these singletons would not contribute to our phylogenetic inferences and might instead create noise in supermatrix analyses. To avoid losing real signal, we do not remove sequences if these sequences fall together with related taxon in unexpected places on the tree.

We had two different approaches to dealing with contamination, depending on our understanding of the taxon. If a taxon was from a transcriptome sequencing project and was known to feed on other eukaryotes, or if a taxon lives in close relationship with another organism (i.e. endosymbiont or parasite), we prescreened the sequences of these taxa by BLAST and retained only those with close identity to the taxon of interest. Many genes for these taxa had no significant BLAST hit – to either the taxon or the potential contaminant – and in this case we may have lost significant amounts of ’real’ data but we felt this was appropriate as we sought to retain sequences with robust evidence of homology across diverse eukaryotes.

In addition to removing sequences from known potential contaminants (e.g. food sources, symbionts), we scanned single gene trees for contaminating sequences in any taxon. For each single gene tree, we looked by script for ‘misplaced’ taxa, i.e. single sequences nested within non-closely related taxa. Here, our naming standard was very useful, as a sequence beginning ‘Ba’ for bacteria should not be nested amongst the ‘Pl’ for Plantae. Using this approach, we found and removed many putatively eukaryotic sequences that were identical to, or deeply nested within, bacterial sequences. This likely caused the removal of recent lateral gene transfers, but these would not contribute to our phylogenetic inferences of vertical descent.

**Trimming datasets for concatenation** **- choosing orthologs not paralogs**.

At this point, we have described how we optimized the number of gene sequences in our alignments from each taxon by removing putative alleles, non-overlapping sequences and contaminants. Still, many taxa will have multiple sequences for some genes, due to paralogy. For some analyses (i.e. gene tree - species tree reconciliation, gene family studies) paralogous sequences are informative but only one paralog per taxon can be used to build the concatenated alignment for super-matrix analyses.

Given the poor resolution at deep nodes in most single gene trees constructed at the scale of all eukaryotes, it is challenging to choose which sequences to retain. Our pipeline builds a maximum likelihood tree with RAxML 19 and uses the topology of the tree to determine which sequences to keep. To maximize retention of orthologous groups, we choose to keep sequences that have the largest numbers of close neighbors from the same clade of eukaryotes. For taxa of unknown phylogenetic position and for those that have an equal number of appropriate neighbors, we choose the shortest branched sequence. So, for example, if *Arabidopsis thaliana* has three putative paralogs for a given gene - one in a clade containing 5 Plantae (green algae, red algae and glaucocystophytes), one in a clade containing 10 Plantae, and one in a clade of fungi - we would choose to retain the sequence in the large Plantae group and remove the others.

**Time and computational** **power**

Gene and taxon-rich analyses are time consuming, and many of the steps in this process require a considerable amount of memory. We ran each step on an 8 core, 16GB iMAC as well as on a high-performance computer cluster with both AMD and Intel processors. We made each step in the pipeline as independent as possible so they can be split up over several computers or nodes on a cluster. We ‘parallelized’ by running our scripts concurrently and then combining the results. In this way, we have done a full phylogenomic analysis in as little as 4 days, from ortholog determination to a final concatenated super-matrix ready for tree building. We have not performed formal run time analyses, and the scripts could, most likely, be made more efficient, including rewriting some of the code to use more efficient data structures. Nevertheless, our pipeline allows us to generate robust alignments in a timely manner.

**Flowchart showing major steps in the pipeline:**
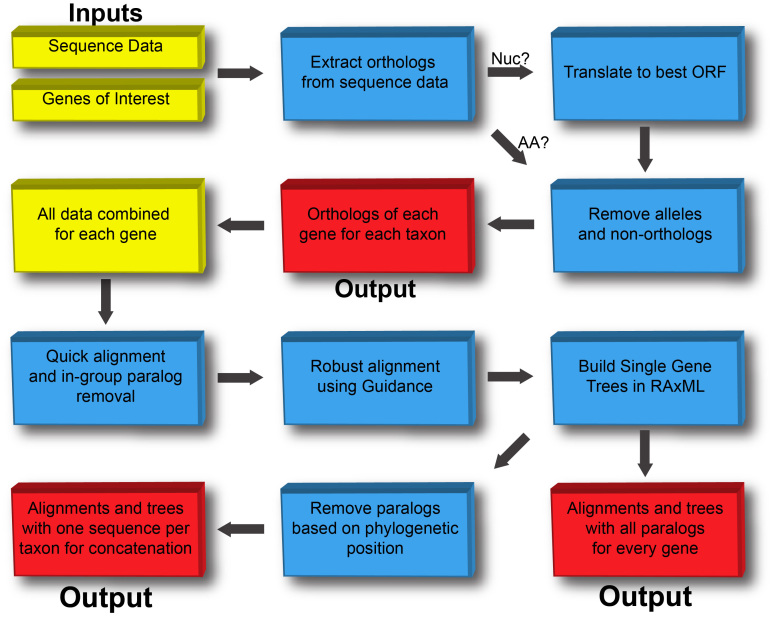
First, the scripts relating to Taxon Objects output orthologs for each gene of interest by starting with sequence data from target taxa and fasta files of orthogs from OrthoMCL. The orthologs are then combined into Gene Objects and a series of refinement steps are performed including removal of ingroup paralogs, alignment with Guidance (Penn et al., 2010) and generation of single gene trees. The outputs are alignments and trees with 1) all paralogs and 2) paralogs removed in preparation for concatenation.

## Part II: A more detailed description of scripts

Scripts are available at https://github.com/Katzlab/Pipeline. In the following section, we describe the pipeline in more detail; when we refer to a specific place in the scripts, we give a comment that will flag the place where the methods being described begin (i.e. “see *Taxon1*” means search for *Taxon1* in Taxon/__init__.py).

The first step, prior to running the pipeline, is to collect data and prepare it for input. The OrthoMCL database was downloaded in its entirety from OrthoMCL (http://www.orthomcl.org/) and renamed according to the naming strategy described above. This includes all sequence data from the genomes used by OrthoMCL, plus their orthologous group assignment. The data from taxa to be added (i.e. sequences from GenBank, or newly sequenced data) were downloaded and renamed as well. This step is critical, as all our scripts rely on taxa being named correctly. A renaming script for fasta files downloaded from GenBank (renamefasta.py) can be found in our github (https://github.com/Katzlab/Pipeline), and can be adapted for data from other sources. As mentioned above, if the taxon is known to have a eukaryotic food source or to have an endosymbiotic or parasitic relationship with another eukaryote, further quality control scripts should be used to clean the data.

Our scripts revolve around a Pipeline Object (Pipeline/__init__.py), which contains the orthologous groups (OGs) of interest, data from OrthoMCL, and sequence data from each taxon in the pipeline (Fig. 1). Each instance of the Pipeline Class takes these inputs and builds many Taxon Objects (Taxon/__init__.py), which mine and refine data from individual taxa. Once all Taxon Objects have finished, the data for each OG are combined into Gene Objects (Gene/__init__.py), from which alignments and phylogenies are built and further refined.

**Taxon Objects**

The series of methods contained in a Taxon Class begins with a fasta file for each taxon, which can be downloaded from public database or collected from newly sequenced data. Methods in the Taxon class are executed so that orthologs are extracted (see *Taxon1*), amino acid sequences are inferred (if the original data are nucleotide sequences; see *Taxon2*) and the data are refined to remove alleles and non-homologous sequences (see *Taxon3*).

In the first step, the sequences from each taxon are analyzed with BLAST against downloaded OrthoMCL data and if a sequence has a significant hit to an OG of interest, it is retained and stored in a file for each taxon/OG combination. Each taxon may have multiple sequences for some or all OGs of interest as these sequences may represent allelic variation, paralogs, identical sequences from different projects, or sequences with shared motifs that are not actually homologous to the gene of interest.

If the original data are nucleotides, the amino acids of the retained sequences are inferred using a series of methods within the Taxon class. Translating into appropriate reading frames is non-trivial, especially for error prone transcriptome projects, ESTs or reads from the Sequence Read Archive of Next Generation Sequences at NCBI, because such data often contain frame shift errors. For each sequence, our Taxon Object scripts first use BLASTx against the best OG hit for that sequence. This step will show if the sequence has one reading frame and, if so, the sequence is translated in that frame. If the BLAST report shows significant hits to multiple reading frames, we assume a frame shift mutation and skip ahead, one nucleotide at a time, until the translation comes back into frame. We may ‘lose’ an amino acid in this process, but we attempt to ensure that the translation is as long and as accurate as possible.

Other methods in the Taxon Object attempt to remove sequences that represent allelic variation or non-homologous sequences. All sequences from a taxon for a given OG are first compared *via* pairwise alignment as implemented with EMBOSS Needle (http://www.ebi.ac.uk/Tools/psa/emboss_needle/). Sequences that are too similar (i.e. alleles of a gene or very recent paralogs) are removed. For our data, we chose to remove the shorter of the two sequences if they were from the same taxon and were more than 98% identical over the region of overlap, though this parameter can be changed (see *Taxon_P1*).

At this point we also remove obvious cases where homology inferences are not robust; two sequences that are sufficiently dissimilar in their pairwise comparison may be motif-sharing proteins that are not orthologous, or they may be non-overlapping sequences. Based on our inspection of numerous test cases, similarity scores of less than 75% from Needle comparisons represent non-homologous or non-overlapping sequences and we removed the shorter of the two. Users may wish to test different parameters here, which can be changed at *Taxon_P1*.

**Gene Objects**

After the sequence data from each Taxon Object have been sorted into OGs, translated and extraneous data have been removed, the data from all taxa per OG are combined. These are the starting point for the Gene Objects, which are passed through another series of data refinement methods. Scripts for the Gene Objects first refine the data by building quick alignments and trees in Mafft 20 and removing all but one sequence from in-group paralogs - sequences from one taxon that form a monophyletic group in the Mafft guide tree (see *Gene1*). These sequences represent recent gene duplications, add little information to a phylogenetic analysis, and increase computational costs. Since we are working here with a fast tree, the script iterates this process several times to remove more sequences that may have been misplaced in previous iterations. For other types of studies, it may be desirable to retain all paralogs, in which case, the in-group paralog removal step can be skipped (See *Gene5* and *Pipeline1*.)

Once the in-group paralogs are removed, the scripts use a stand-alone version of Guidance 21 to build a higher quality alignment. Guidance builds alignments, scores each taxon and each character position in the alignment, and allows for removal of taxa and/or positions that do not meet the score criteria. The standard criteria for Guidance were too strict for our extremely diverse dataset so we modified them through inspection to suit our needs. These parameters were changed in the Perl script for Guidance itself. Specifically, we changed the {SP_SEQ_CUTOFF} and {SP_COL_CUTOFF} to 0.5 and 0.4, respectively, which loosens the stringent criteria for column and sequence scores. For studies that are looking at more closely related organisms, the standard {SP_SEQ_CUTOFF} and {SP_COL_CUTOFF} of 0.6 and 0.93, respectively, might be preferable. After removing taxa with poorly aligned sequences, we align in Guidance again to remove ambiguously aligned characters. The output of this step is a robust alignment with ambiguously aligned taxa and positions removed.

To identify obvious contaminants, the script builds a maximum likelihood tree in RAxML 19 from the robust alignments for each OG and scans these trees for signs of misplaced taxa (see *Gene3*). For our broad taxonomic study, we only remove eukaryotic sequences that are nested within archaea or bacteria, or vice versa. For studies of smaller time scale, it could be useful to have more stringent requirements, such as requiring clades with known relationships to be monophyletic. After the removal of contaminated sequences, the alignments are ready for gene tree/species tree reconciliation analysis, or other methods of analysis that retain all paralogs from all taxa.

To prepare for concatenation and super-matrix analysis, only one sequence per taxon can be retained. Taking the alignments and trees built previously, our script uses a series of methods to scan the tree for multiple sequences from each taxon and then scores each sequence based on its close neighbors on the tree (see *Gene4*). The aim is to retain the sequence in the largest group of related sequences, again taking advantage of our tightly controlled naming strategy. This portion of the script uses the Python module dendropy (http://pythonhosted.org/DendroPy/) to traverse the tree and identify these contaminants.

The final product of the Gene Object scripts are two sets of alignments for each OG – one with paralogs retained for gene tree-species tree reconciliation analyses and one with paralogs removed, ready for concatenation and super-matrix analyses.

## Part III: A case study placing five amoeboid taxa on the tree of life

As an example of the analyses that can be done with this pipeline, we present the transcriptomes from five putative Amoebozoa: *Trichosphaerium* sp., *Pessonella* sp., Eukaryota sp. JRG-2011 (an amoeboid taxon deposited as ‘*Sexangularia* sp. ATCC 50979’ at the American Type Culture Collection and which is being described elsewhere), *Filamoeba nolandi*, and *Stereomyxa ramosa*. Previously, these taxa had few sequences available on GenBank and had been placed in the Amoebozoa by morphology or by the phylogenetic analysis of SSU and a few protein-coding genes. The transcriptomes for these taxa were characterized as part of the Gordon and Betty Moore Foundation’s Marine Microbial Eukaryote Transcriptome Sequencing Project (http://www.moore.org/). We analyzed assembled transcriptome sequence data for these amoeboid taxa.

**Table 1: Transcriptome statistics for Amoebozoa used in case study.** 

Taxon	ATCC	Contigs	SSU	Bacterial	Eukaryotic	Unknown	Genes
*Filamoeba nolandi*	50430	15245	17	2409	12205	7057	171
* Pessonella* sp.	PRA29	17104	31	1783	8695	6626	199
* Trichosphaerium* sp.	40318	22137	15	2252	9541	10344	35
Eukaryota sp. JRG-2011	50979	14754	33	1940	7303	5478	178
*Stereomyxa ramosa*	50982	21354	38	2141	9854	9321	210

Table 1 Notes: Contigs = number of assembled contigs from the Moore Transcriptome Project assembly (http://www.moore.org/marine-micro.aspx). SSU = number of SSU contigs removed from transcriptome data; bacterial = number of contigs with best hits to bacteria (or, rarely, archaea), eukaryotic = number of contigs with robust hits to eukaryotes, unknown = number of contigs with no significant BLAST hit (e.g. are potentially taxon-specific) as determined by custom quality control scripts described in Grant et al. 22. Genes = the number of the potential 328 genes identified for phylogenomic analyses after running the pipeline to completion

Prior to launching the scripts described above, we took care to enhance the quality of the transcriptome data as all of these amoebae are cultured in the presence of diverse bacteria, and failure to remove bacterial contaminants will confound phylogenetic inferences. For each taxon, the assembled contigs that we received from the Moore foundation were passed through quality control scripts as described in Grant *et. al*. 22 to remove ribosomal DNA and bacterial contamination. Retained sequences were used as input for the taxon and gene object scripts described above. We chose 373 OGs from OrthoMCL DB that had representatives from four or five major clades of eukaryotes (including Amoebozoa) and had data from at least 100 taxa. We passed the transcriptome data from the five amoebae, along with data from GenBank, through the pipeline on the University of Florida computer cluster to extract and align homologs for these 373 groups. From the amoeba transcriptomes we retained 171 sequences from *Filamoeba nolandi*, 178 from Eukaryota sp. JRG-2011, 210 from *Stereomyxa ramosa*, 35 from *Trichosphaerium* sp. and 199 from *Pessonella* sp. (Table 1). It is notable that *Trichosphaerium* sp. is only found in 35 of the 373 OGs, even though the number of eukaryotic contigs in the transcriptome is comparable to the other taxa (Table 1). *Trichosphaerium* sp. has very high rates of evolution 23 and it may be that our tools failed to properly assign orthology for many of the genes from this taxon. As with any high throughput process, unexpected results should be examined. There were 45 OGs of the original 373 that did not contain data for the newly added taxa, so we removed these from further analyses, leaving 328 OGs (Supplemental Table S2).

We then employed our scripts to remove potential contaminants (i.e. single taxa nested in distantly related clades), identifying 838 sequences that were nested among sequences from a different domain (e.g. a single eukaryote among bacteria). Among the sequences removed there were 117 Archaea, 238 Bacteria, 10 cryptophytes, 7 haptophytes, 1 kathablepharid, 60 Excavata, 174 Opisthokonts, 110 Plantae, 88 SAR and 23 Amoebozoa (Supplemental Table S3). Only 4 sequences were identified as potential contaminants from the transcriptome data added here, with the rest coming from GenBank sequences. This supports our method of quality control prior to launching the pipeline 22 and the importance of taking care when using data from publicly available sources.

We appended an SSU alignment that contained nearly all of the taxa from GenBank to maximize the completeness of the dataset (see Supplemental Table S4). After concatenation of 238 proteins plus ssu-rDNA, we removed taxa containing fewer than 20 target genes and masked sites with more than 50% missing data. For the final analyses, we also removed the long branched archamoebae (e.g. *Entamoeba* spp.) and microsporidia taxa 24 ^,^ 25 ^,^ 26 ^,^ 27 as well as bacterial and archaeal taxa. The final alignment had 247 diverse eukaryotes and 15,650 characters, from which we built a maximum likelihood tree of the concatenated data in RAxML version 7.3.2 19 ^,^ 28 with rapid bootstrapping followed by a thorough maximum likelihood search as implemented for RAxML’s default parameters. For this analysis we partitioned our data and used GTRGAMMA for the DNA partition and PROTGAMMALG for the proteins as this is most often found to be the best fitting model by ProtTest 29 in our preliminary analyses.

The newly added transcriptomes all fall within the monophyletic Amoebozoa, supporting previous hypotheses about their evolutionary placement (Fig. 2; Fig. 3), although the taxon sampling here doesn’t allow for the analysis of relationships within the Amoebozoa 23 ^,^ 30 ^,^ 31. The resulting tree is generally concordant with most large-scale phylogenetic trees (Fig. 2). We recover the major eukaryotic clades Opisthokont, SAR and Amoebozoa, with 100%, 100% and 93% bootstrap support, respectively. The more controversial group Excavata is non-monophyletic in our analysis, likely due to the placement of some orphan taxa that are unstable in many analyses (e.g.*Subulatomonas*+*Breviata *and *Collodictyon*). However, many of the subclades of Excavata (i.e. Fornicata, Euglenozoa, Heterolobosea, Jakobida) are recovered with full support (See Supplemental Figure S2). The Plantae are also non-monophyletic in this analysis, as the Glaucocystophytes fall outside of a monophyletic group made up of the red and green algae and the proposed Hacrobia 32 : Cryptophytes, Haptophytes, Katablepharids plus*Telonema*).

**Most likely tree of concatenated post-pipeline alignments:**
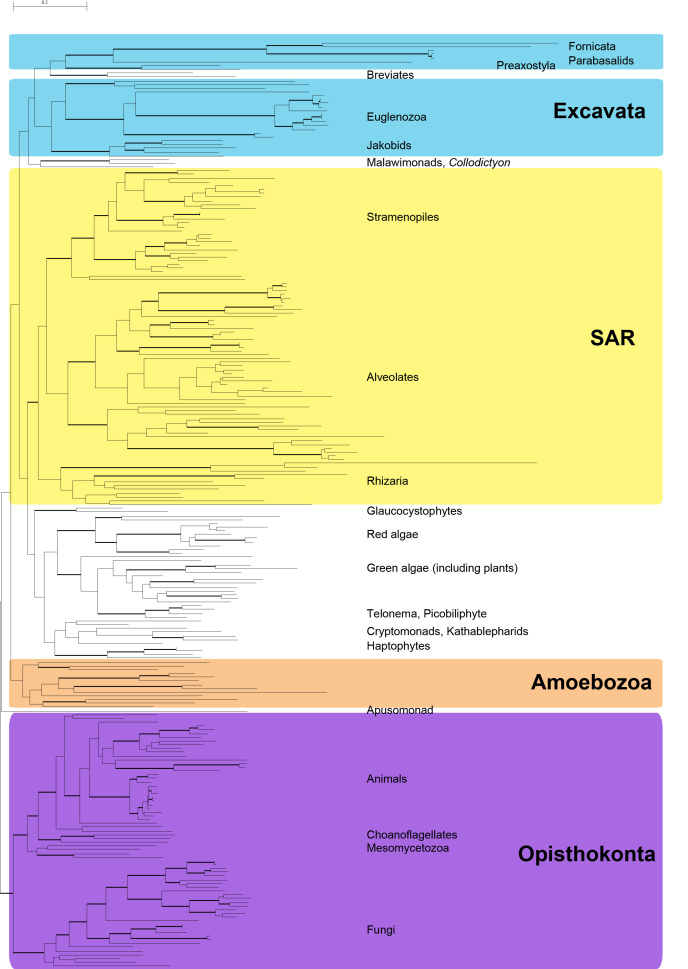
Most likely tree reconstructed using RAxML 7.3.2 with 247 taxa and 15,650 characters (SSU + 238 protein genes). Bold branches have 100% bootstrap support. Tree with support values and branches labeled can be found in the supplemental data (Figure S1).

**Details of Amoebozoa clade with new taxa in bold:**
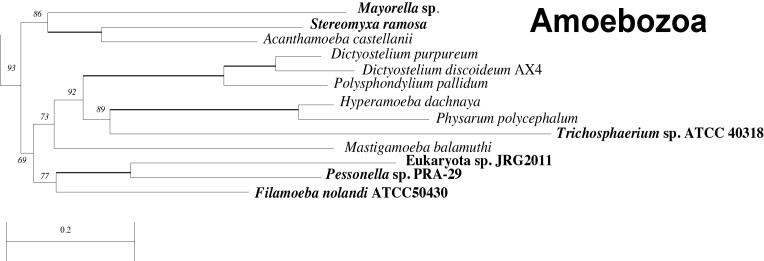
Close-up from the phylogenetic analysis showing the placement of the newly added taxa (in bold) within the Amoebozoa. Nodes labeled with bootstrap support values except for bold branches which have 100% bootstrap support. See Figure 2 for further notes.

## Conclusions

The scripts developed for this study and the discussion of our approach will be helpful to anyone wanting to begin large-scale phylogenomic analyses. In addition, our approach allowed for the placement of newly sequenced amoebozoa taxa on the tree of life, filling in a fairly sparse region on the eukaryotic tree of life.

## Availability of supporting data

All transcriptome sequence data from the Gordon and Betty Moore Foundation are available (http://camera.crbs.ucsd.edu/mmetsp/list.php). Our final alignment and tree are accessible through TreeBase (http://purl.org/phylo/treebase/phylows/study/TB2:S14480) and all scripts will be available through the following github repository: https://github.com/Katzlab/Pipeline. Supplemental data files are available at figshare.com ( dx.doi.org/10.6084/m9.figshare.953176)

## Competing Interest Statement

The authors declare that no competing interests exist.

## Figures and Tables

**Figure:**
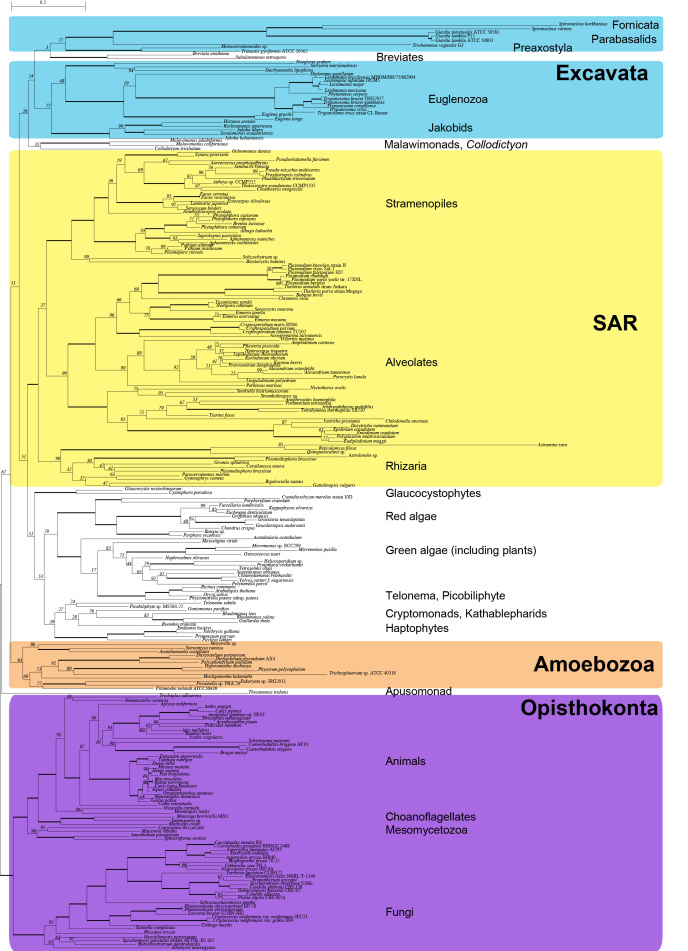
Most likely tree reconstructed using RAxML 7.3.2 with 247 taxa and 15,650 characters (SSU + 238 protein genes). Bold branches have 100% bootstrap support, other support values are labeled.

## References

[1] Katz LA, Grant JR, Parfrey LW, Gant A, O'Kelly CJ, Anderson OR, Molestina RE, Nerad T: Subulatomonas tetraspora nov. gen. nov. sp. is a member of a previously unrecognized major clade of eukaryotes. Protist 2011, 162(5):762-773. 10.1016/j.protis.2011.05.002 21723191

[2] Zhao S, Burki F, Brate J, Keeling PJ, Klaveness D, Shalchian-Tabrizi K: Collodictyon--an ancient lineage in the tree of eukaryotes. Mol Biol Evol 2012, 29(6):1557-1568. 10.1093/molbev/mss001 PMC3351787 22319147

[3] Kudryavtsev A, Pawlowski J: Squamamoeba japonica n. g. n. sp (Amoebozoa): A Deep-sea Amoeba from the Sea of Japan with a Novel Cell Coat Structure. Protist 2013, 164(1):13-23. 10.1016/j.protis.2012.07.003 22964370

[4] Kudryavtsev A, Pawlowski J, Hausmann K: Description of Paramoeba atlantica n. sp (Amoebozoa, Dactylopodida) - a Marine Amoeba from the Eastern Atlantic, with Emendation of the Dactylopodid Families. Acta Protozool 2011, 50(3):239-253.

[5] Hampl V, Hug L, Leigh JW, Dacks JB, Lang BF, Simpson AGB, Roger AJ: Phylogenomic analyses support the monophyly of Excavata and resolve relationships among eukaryotic "supergroups". Proc Natl Acad Sci U S A 2009, 106(10):3859-3864. 10.1073/pnas.0807880106 PMC2656170 19237557

[6] Parfrey LW, Grant J, Tekle YI, Lasek-Nesselquist E, Morrison HG, Sogin ML, Patterson DJ, Katz LA: Broadly sampled multigene analyses yield a well-resolved eukaryotic tree of life. Syst Biol 2010, 59(5):518-533. 10.1093/sysbio/syq037 PMC2950834 20656852

[7] Katz LA, Grant JR, Parfrey LW, Burleigh JG: Turning the crown upside down: gene tree parsimony roots the eukaryotic tree of life. Syst Biol 2012, 61(4):653-60. 10.1093/sysbio/sys026 PMC3376375 22334342

[8] Dunn CW, Howison M, Zapata F: Agalma: an automated phylogenomics workflow. Bmc Bioinformatics 2013, 14 10.1186/1471-2105-14-330 PMC3840672 24252138

[9] Robbertse B, Yoder RJ, Boyd A, Reeves J, Spatafora JW: Hal: an automated pipeline for phylogenetic analyses of genomic data. PLoS Curr 2011, 3:RRN1213 10.1371/currents.RRN1213 PMC3038436 21327165

[10] Rodriguez-R LM, Grajales A, Arrieta-Ortiz ML, Salazar C, Restrepo S, Bernal A: Genomes-based phylogeny of the genus Xanthomonas. Bmc Microbiology 2012, 12. 10.1186/1471-2180-12-43 PMC3359215 22443110

[11] Peters RS, Meyer B, Krogmann L, Borner J, Meusemann K, Schutte K, Niehuis O, Misof B: The taming of an impossible child: a standardized all-in approach to the phylogeny of Hymenoptera using public database sequences. Bmc Biol 2011, 9 10.1186/1741-7007-9-55 PMC3173391 21851592

[12] Smith SA, Beaulieu JM, Donoghue MJ: Mega-phylogeny approach for comparative biology: an alternative to supertree and supermatrix approaches. BMC Evol Biol 2009, 9:37 10.1186/1471-2148-9-37 PMC2645364 19210768

[13] Knoll AH: Proterozoic and early Cambrian protists: evidence for accelerating evolutionary tempo. Proc Natl Acad Sci 1994, 91:6743-6750. 10.1073/pnas.91.15.6743 PMC44278 8041692

[14] Parfrey LW, Lahr DJG, Knoll AH, Katz LA: Estimating the timing of early eukaryotic diversification with multigene molecular clocks. Proc Natl Acad Sci U S A 2011, 108(33):13624-13629. 10.1073/pnas.1110633108 PMC3158185 21810989

[15] Ostlund G, Schmitt T, Forslund K, Kostler T, Messina DN, Roopra S, Frings O, Sonnhammer ELL: InParanoid 7: new algorithms and tools for eukaryotic orthology analysis. Nucleic Acids Res 2010, 38:D196-D203 10.1093/nar/gkp931 PMC2808972 19892828

[16] Li L, Stoeckert CJ, Jr., Roos DS: OrthoMCL: Identification of Ortholog Groups for Eukaryotic Genomes. Genome Research 101101/gr1224503 2003, 13(9):2178-2189 10.1101/gr.1224503 PMC403725 12952885

[17] Knoll AH, Javaux EJ, Hewitt D, Cohen P: Eukaryotic organisms in Proterozoic oceans. Philos Trans R Soc B-Biol Sci 2006, 361(1470):1023-1038 10.1098/rstb.2006.1843 PMC1578724 16754612

[18] Altschul SF, W. Fish, W. Miller, E.W. Myers, and D.J. Lipman: Basic local alignment search tool. J Mol Biol 1990, 215:403-410. 10.1016/S0022-2836(05)80360-2 2231712

[19] Stamatakis A: RAxML-VI-HPC: Maximum likelihood-based phylogenetic analyses with thousands of taxa and mixed models. Bioinformatics 2006, 22(21):2688-2690. 10.1093/bioinformatics/btl446 16928733

[20] Katoh K, Asimenos G, Toh H: Multiple Alignment of DNA Sequences with MAFFT. Bioinformatics for DNA Sequence Analysis 2009, 537:39-64. 10.1007/978-1-59745-251-9_3 19378139

[21] Penn O, Privman E, Ashkenazy H, Landan G, Graur D, Pupko T: GUIDANCE: a web server for assessing alignment confidence scores. Nucleic Acids Res 2010, 38:W23-W28 10.1093/nar/gkq443 PMC2896199 20497997

[22] Grant JR, Lahr D. J. G, Rey F, Gordon JI, Knight R, Molestina RE, Katz LA: Gene discovery in the transcriptomes of three diverse microbial eukaryotes: Corallomyxa tenera, Chilodonella uncinata, and Subulatomonas tetraspora. Protist Genomics 2012, 1:3-18.

[23] Tekle YI, Grant J, Anderson OR, Nerad TA, Cole JC, Patterson DJ, Katz LA: Phylogenetic placement of diverse amoebae inferred from multigene analyses and assessment of clade stability within 'Amoebozoa' upon removal of varying rate classes of SSU-rDNA. Mol Phylogenet Evol 2008, 47(1):339-352. 10.1016/j.ympev.2007.11.015 18180171

[24] Keeling PJ, Fast NM, Law JS, Williams BaP, Slamovits CH: Comparative genomics of microsporidia. Folia Parasitol (Praha) 2005, 52(1-2):8-14. 10.14411/fp.2005.002 16004359

[25] Keeling PJ, Fast NM: Microsporidia: Biology and evolution of highly reduced intracellular parasites. Annu Rev Microbiol 2002, 56:93-116. 10.1146/annurev.micro.56.012302.160854 12142484

[26] Dong JH, Wen JF, Xin DD, Lu SQ: Evolutionary status of Entamoeba. Chinese Sci Bull 2004, 49(17):1847-1853.

[27] Yoon HS, Grant J, Tekle YI, Wu M, Chaon BC, Cole JC, Logsdon JM, Patterson DJ, Bhattacharya D, Katz LA Broadly sampled multigene trees of eukaryotes. BMC Evol Biol 2008, 8(14)10.1186/1471-2148-1188-1114. 10.1186/1471-2148-8-14 PMC2249577 18205932

[28] Stamatakis A, Hoover P, Rougemont J: A rapid bootstrap algorithm for the RAxML web-servers. Syst Biol 2008, 57:758-771. 10.1080/10635150802429642 18853362

[29] Abascal F, Zardoya R, Posada, D. 2005. ProtTest: Selection of best-fit models of protein evolution. Bioinformatics: 21(9):2104-2105. 10.1093/bioinformatics/bti263 15647292

[30] Smirnov A, Nassonova E, Berney C, Fahrni J, Bolivar I, Pawlowski J: Molecular phylogeny and classification of the lobose amoebae. Protist 2005, 156(2):129-142. 10.1016/j.protis.2005.06.002 16171181

[31] Lahr DJG, Grant J, Nguyen T, Lin JH, Katz LA: Comprehensive phylogenetic reconstruction of Amoebozoa based on concatenated analyses of SSU-rDNA and actin genes. PLoS One 2011, 6(7). 10.1371/journal.pone.0022780 PMC3145751 21829512

[32] Yoon HS, Price DC, Stepanauskas R, Rajah VD, Sieracki ME, Wilson WH, Yang EC, Duffy S, Bhattacharya D: Single-Cell Genomics Reveals Organismal Interactions in Uncultivated Marine Protists. Science 2011, 332(6030):714-717. 10.1126/science.1203163 21551060

