# Correction: Building a Phylogenomic Pipeline for the Eukaryotic Tree of Life - Addressing Deep Phylogenies with Genome-Scale Data

**DOI:** 10.1371/currents.tol.e089df47766ed1e9dabac39c76bae266

**Published:** 2015-03-06

**Authors:** PLOS Currents

## Correction

In table 1 an incorrect value was provided for the "Contigs" value for "*Filamoeba nolandi*" (Column 3, row 2). The corrected table is provided below:

**Table 1: Transcriptome statistics for Amoebozoa used in case study.** 

Taxon	ATCC	Contigs	SSU	Bacterial	Eukaryotic	Unknown	Genes
*Filamoeba nolandi*	50430	21671	17	2409	12205	7057	171
* Pessonella* sp.	PRA29	17104	31	1783	8695	6626	199
* Trichosphaerium* sp.	40318	22137	15	2252	9541	10344	35
Eukaryota sp. JRG-2011	50979	14754	33	1940	7303	5478	178
*Stereomyxa ramosa*	50982	21354	38	2141	9854	9321	210

## Reference

Grant JR, Katz LA. Building a Phylogenomic Pipeline for the Eukaryotic Tree of Life – Addressing Deep Phylogenies with Genome-Scale Data. PLOS Currents Tree of Life. 2014 Apr 2. Edition 1. doi: 10.1371/currents.tol.c24b6054aebf3602748ac042ccc8f2e9.

View Article

